# Improved YOLOv8 for early detection of Alzheimer’s disease from magnetic resonance imaging

**DOI:** 10.3389/fnagi.2026.1771536

**Published:** 2026-07-29

**Authors:** Benedictor Alexander Nguchu, Jin Han, Dennis Nestory Mwighusa, Li Li, Kaile Su, Peter Shaw

**Affiliations:** 1 Oujiang Laboratory (Zhejiang Lab for Regenerative Medicine, Vision and Brain Health), Wenzhou Medical University, Wenzhou, Zhejiang, China; 2 School of Computer and Control Engineering, Yantai University, Yantai, China; 3 Africa Research Institute For AI (ARIFA), Dar es Salaam, Tanzania; 4 College of Electronic Engineering, Tianjin University of Technology and Education, Tianjin, China

**Keywords:** Alzheimer’s disease, attention mechanism, MRI medical images, object detection, YOLOv8 network

## Abstract

**Background:**

Alzheimer’s disease (AD) is a life-threatening condition affecting 47 million people globally, with 13% of them over the age of 65. Despite ongoing efforts in drug development, the incidence of Alzheimer’s disease is increasing, projected to reach 131 million new cases in the next two decades. Delayed diagnosis and failure to uncover the neuropathological pathways of AD contribute to the increasing incidence and sequelae of AD.

**Methods:**

Here, we address these challenges by developing a model derived from YOLOv8 for the early diagnosis of AD. We improve YOLOv8 by replacing the CBS convolution modules with the RepVGG (Reparameterized VGG) module and appending the Spatial Pyramid Pooling Enhanced with ELAN (SPPELAN) module and Simple Attention Module (SimAM) at the end of the YOLOv8 network. Additionally, we improve the model by introducing C2f_EMA module at YOLOv8 neck.

**Results:**

Our results demonstrate a significant improvement in our model’s performance compared to the benchmark YOLOv8 for AD detection. While the benchmark YOLOv8 showed performance metrics of 0.794 accuracy, 0.876 recall, 0.881 mAP50, and 0.875 mAP50:95, our model demonstrated improved performance with 0.816 accuracy, 0.877 recall, 0.904 mAP50, and 0.898 mAP50:95, indicating improvements of 2.77, 0.11, 2.61, and 2.62%, respectively. These findings provide insights into the possibility of achieving an effective diagnosis at the early stage of AD, which may aid early personalized intervention and further assist researchers in conducting in-depth examinations to understand the early mechanisms underlying AD.

## 1 Introduction

Alzheimer’s disease (AD) is a life-threatening neurodegenerative condition characterized by a gradual decline in cognitive function (Wilson et al., 2012), affecting people usually those over 65 years of age (Huff et al., 1987; Oh et al., 2024). By 2019, there were about 50 million people in the global who were reported by WHO to have AD (Global Burden of Disease (GBD) dataset) (Javaid et al., 2021). By 2050, the number of AD cases is projected to triple to 150 million (Stavropoulos et al., 2020). Despite the increasing threat from AD, with skyrocketing incidences yearly, the development of its therapeutics is still lagging behind. We hypothesize that two reasons may be behind this delay in drug development, i.e., late diagnosis and a lack of a full understanding of the neuropathological pathways of AD. In most cases, subjects or patients with AD are clinically diagnosed when the disease is already at a mild or advanced stage, when large parts of molecular and cellular mechanisms affected, making it harder to understand the personalized pathways leading to the condition.

On the other hand, recent studies have shown that our understanding of the neuro-underpinnings of AD is still in question, as new discoveries challenge early beliefs about the pathogenesis of AD. For example, AD has long been thought to be primarily caused by abnormalities in amyloid *β* (Aβ) and tau. However, recent studies suggest otherwise; the pathogenesis of AD is far more complex than it was thought in earlier hypotheses, with the new theories and evidence challenging the traditional “single cause” view. Contrary to just neurotoxicity caused by Aβ and tau, the growing evidence point out that AD is multifactor-driven disease (Sorbi and Ferrari, 2021; Wong-Guerra et al., 2023), with chronic neuroinflammation, immune system abnormalities, dysfunction of microglia and astrocytes, and alterations in the gut-brain axis all playing an important role in the pathogenesis. The mutations in genes such as APP, PSEN1, and PSEN2, which were thought to be the main genetic cause of AD are now found to have a very small influence to the AD cases (Checler and Turner, 2012; Rogaev et al., 1995; Schellenberg and Montine, 2012). The new evidence suggests that AD risk may be more associated with completely new and multiple genes, which may include ABCA7, BIN1, CD33 (Checler and Turner, 2012; Rogaev et al., 1995; Schellenberg and Montine, 2012). Peripheral amyloid dysfunction and infection can also induce the production of Aβ and tau as antimicrobial peptides, resulting in immune response activation and nerve damage (Nelson, 2022; Wong-Guerra et al., 2023). This new peripheral-induced amyloid challenges the traditional view that AD results solely from endogenous lesions of the central nervous system. Proteomics and bioinformatics (Yadikar et al., 2024) identify that early-stage AD is also associated with abnormal molecular pathways, including mitochondrial dysfunction, synaptic vesicle pathways, and neuro-inflammatory signaling. These abnormalities involve energy metabolism, synaptic function, and the cytoskeleton. These new directions also suggest that AD may result from specific epigenetic changes in neurons, which may include DNA methylation and hydroxymethylation (Fetahu et al., 2019).

Considering these complexities in AD, it is without doubt that there is a need to invest in more research to understand AD and facilitate early diagnosis and clinical interventions. Therefore, here we attempt to contribute in on-going AD research by developing a model for early detection of AD from MRI perspective. We built our model from YOLOv8 architecture with architectural innovations in the backbone and neck networks. First, we replace the CBS convolution modules with the RepVGG (Reparameterized VGG) module and append the Spatial Pyramid Pooling Enhanced with ELAN (SPPELAN) module and Simple Attention Module (SimAM) at the backbone network of YOLOv8 architecture. Second, we introduce C2f_EMA module at YOLOv8 neck network to replace C2f. Our team believes that our architectural modifications would provide a more effective diagnostic tool for AD. The overall detection of AD at an early stage is essential for understanding the initial pathways and mechanisms of AD pathology, guiding researchers toward discovering proper therapies.

It is important to note that there is a gap between the ongoing brain changes and the clinical diagnosis of AD. While the clinical diagnosis of AD is usually positive at least 3 years later (Soldan et al., 2015; Younes et al., 2014), studies indicate that brain atrophy begins earlier than that. The preliminary evidence suggests that, at early stages before clinical manifestations, AD is accompanied with atrophy in the medial temporal lobe (Kwak et al., 2022; Scahill et al., 2002; Whitwell et al., 2007). This region include areas like amygdala, anterior hippocampus, and entorhinal cortex (Kwak et al., 2022; Scahill et al., 2002; Whitwell et al., 2007). At this stage, most changes are subtle and nearly impossible to detect with conventional methods. When clinical tests show positive for AD, the level of brain atrophy is already severe and advanced. By then, several brain regions exhibit significant atrophy as atrophy expands to wide areas, including the temporal, parietal, occipital, and frontal cortex. Interestingly, before this stage, i.e., in the prodromal phase of AD, the spread and severity of atrophy vary greatly from person to person (Ferreira et al., 2017). The variation in clinical symptoms and levels of atrophy is even intensified between subjects with early-onset AD and those with late-onset AD (Möller et al., 2013). At a late stage, which is often when patients are presented for clinical tests due to difficulties and health challenges in their daily activities, most patients exhibit similar advanced symptoms as atrophy has spread throughout the brain regions due to the severity of the disease (Scahill et al., 2002; Whitwell et al., 2007). At this stage, understanding the underlying generalized and personalized mechanisms of AD becomes nearly impossible, as several biological processes are already impaired. In light of this, MRI-based early detection of AD is crucial for a better understanding of the disease and possible tailoring of timely intervention before the disease progresses or before clinical symptoms manifest.

There are considerable work conducted previously to detect AD using Machine Learning and AI models. The recurrent neural network (RNN) and convolutional neural network (CNN) are among these models. The former has been used to capture the structural/temporal aspects while the latter has been used to capture the levels of amyloid plaque accumulation to predict the progression of AD (Tang et al., 2018; Yoon et al., 2021). For example, Tang et al. (2018) developed a CNN-architecture-based pipeline to identify amyloid plaque and visualize pathological distribution in immunohistochemical sections (Tang et al., 2018). From MRI perspective, one report also caught our attention (Mahjoubi et al., 2024). In this report, the authors identify and classify an MRI image of AD patients from healthy controls. With this model, the authors achieved an accuracy of 98%. Other teams have also been working on CNN models for AD diagnosis using different imaging modalities besides MRI. For example, Tufail et al. (2022) leveraged CNN architecture on PET images for early-stage AD categorization (Tufail et al., 2022). They specifically applied CNN models in both 2D and 3D domains to classify patterns of the initial stages of AD from mild cognitive impairment (MCI) and normal controls(NC), using both binary and multiclass classification approaches. The authors reported a performance of 59.73% with a 3D-CNN architecture on multiclassification tasks (AD vs. MCI vs. NC). While these models show promise to some extent, they still indicate inconsistency and poor performance in some cases. In addition to that, there is still a fundamental challenge in how they address the AD detection issue. In most cases, AD identification using CNN is treated as a classification problem, which focuses more on the overall features of an image from one class that can be distinctive from the overall features of an image from another class. Even when AD detection in CNN models is treated purely as a detection problem, it is always considered a two-stage problem rather than a one-stage problem. This way of treatment is computationally cost and makes the model parameters overly intensive.

To address these challenges in AD detection, this study proposes and emphasizes a shift in focus from a two-stage problem to a one-stage problem. This will not only speed up the learning process of the model but also reduce computation costs while improving detection performance (Diwan et al., 2022; Sirisha et al., 2023). A Single or one-stage models have recently been highly endorsed for precise diagnosis (Ćirković and Stanić, 2024; Hamed et al., 2020; Salman et al., 2022). The most popular one-stage models are the YOLO (You Only Look Once) models. The YOLO model series has been widely used in image processing, particularly in image recognition (Guan, 2023; Z. Zhang, 2023), where it has proven useful and effective. Its effectiveness comes from high efficiency in computation time and accuracy. Studies comparing the YOLO series with other models have shown that its capabilities surpass those of the most traditional two-stage detection models like RNN (Ahmed et al., 2024; Cheng et al., 2021). Building on this, we aim to explore the utility of the YOLO series in the early detection of AD. We specifically focus on proposing an improved version of YOLOv8, which we develop by introducing new aspects in the backbone and neck networks of the YOLOv8 architecture. The improved model from this study is expected to serve as a diagnostic tool for early AD in real-time data. Upon achieving high performance, the model can be deployed for clinical applications as an early screening tool for AD, offering a chance for early clinical intervention.

## 2 Materials and methods

### 2.1 YOLOv8 network principle

YOLOv8 is one of the latest versions of the YOLO model series for object detection. YOLO models are named after the concept of “You Only Look Once,” first proposed by Redmon et al. (2015) and Terven et al. (2023). The key innovation of YOLO is its ability to address the object detection task as a single neural network regression problem. This innovation has greatly improved the speed of detection and the overall efficiency. Prior to YOLOv8, numerous improvements have been made to optimize earlier versions of YOLO technology—YOLOv1 to YOLOv7—in terms of network structure, feature extraction, detection accuracy, and speed (Betti and Tucci, 2023; Lee and Hwang, 2021; Li et al., 2023; Li et al., 2021; Terven et al., 2023; Tian et al., 2024; Xiao et al., 2024; Xu and Wu, 2021; Yue and Meng, 2024). YOLOv8 itself is the outcome of the modifications of previous models. The key breakthroughs made were mainly involving the network structure and optimization techniques. YOLOv8 abandons the traditional anchor-frame and adopts an anchorless free detection head, making it structurally simple, and highly flexible for detection (Kumar et al., 2024; Liu et al., 2024; Yaseen, 2024). The model is no longer relying on predefined anchor boxes to predict the location and size of the target. It directly regresses the center point and bounding box size of the target.

This version of YOLO incorporates various multi-scale feature fusion technologies, which significantly enhances the YOLO ability to detect targets at different resolutions, scales and sizes. This makes YOLOv8 a favorite for complex scenes and small targets. The most notable fusion technologies and other technologies that can be employed in YOLOv8 to enhance its capability include multi-scale attention mechanisms, lightweight fusion modules, and bidirectional feature pyramid networks (BiFPN) (or weighting bidirectional feature pyramid networks (WBiFPN)) (Du et al., 2024; Shen et al., 2024; Wang and Jia, 2024; Yu et al., 2024; Zhao et al., 2024). For example, the Efficient Multi-Scale Attention, SimAM (Simple Attention Module), Self-Calibrating Shuffle Attention, and Multi-Scale Convolutional Attention are some of YOLOv8’s fusion technologies based on attention mechanisms. The other technologies are those based on lightweight multi-scale fusion and include techniques such as Multi-Scale Fusion Lightweight Neck, LMSMC (Lightweight Multi-Scale Mixed Convolution), GIFM (Global Information Fusion Module), and FLCM (Four-Leaf Clover Fusion Module). Other technologies involve integrating spatial pyramid pooling (SPP) and BiFPN in the same YOLO framework. This strategy is usually implemented by introducing SPP after the backbone network to enhance feature expression while performing multi-layer feature fusion through BiFPN.

### 2.2 YOLOv8 basic network

**Figure 1:**
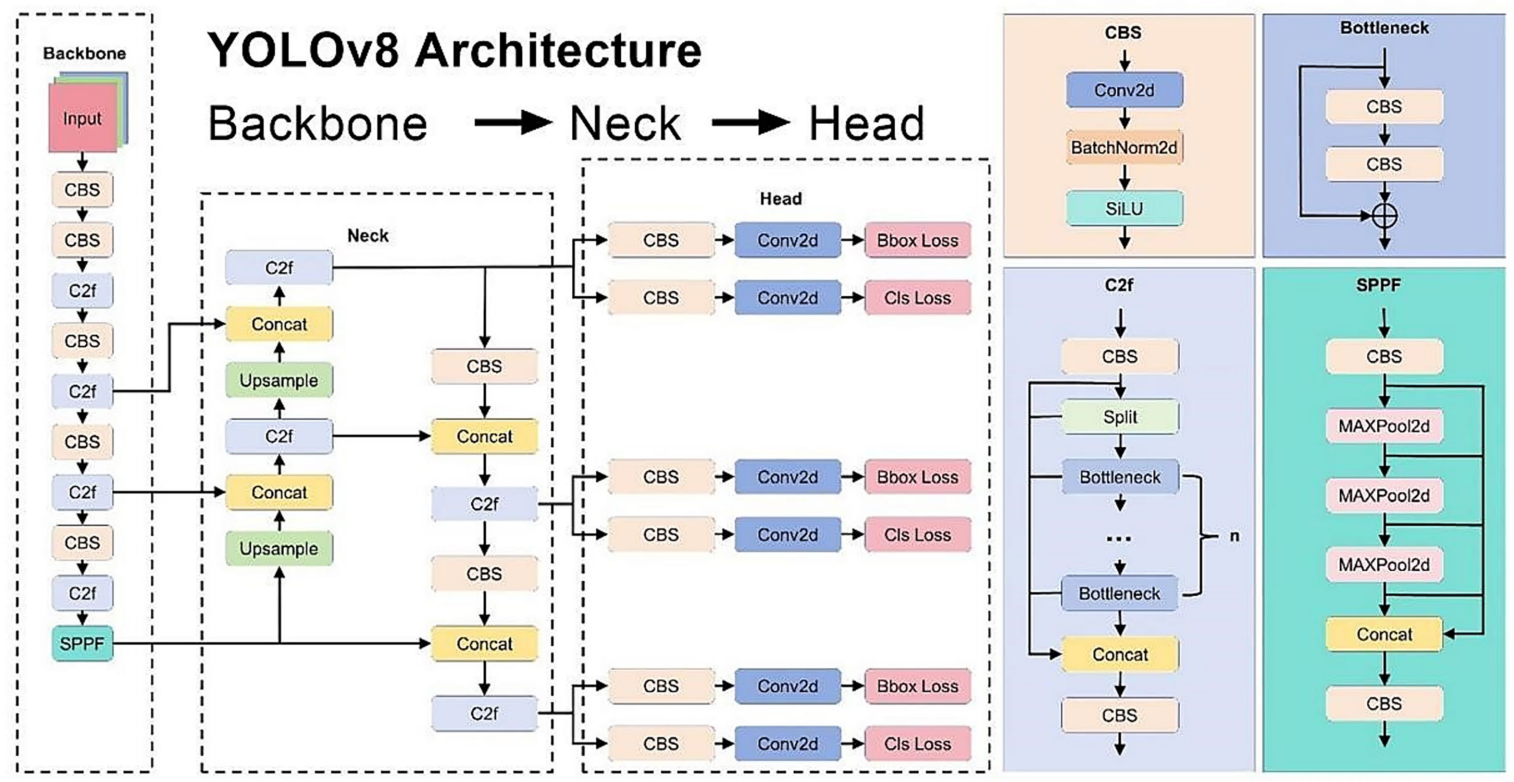
The Network Architecture of Canonical YOLOv8. There are three parts that form the YOLOv8, the Backbone, Neck, and Head. The backbone (left panel) is made of a set of convolutional layer modules (CBS, optimized by batch normalization and SiLU activation function), C2f Module, and Fast Spatial Pyramid Pooling Module (SPPF). The CBS stands for 2-dimensional convolution layer (Conv2d), with a batch normalization layer (BatchNorm2d), and a SiLU activation function. This part is responsible for performing convolution operations on feature maps and convolution kernels. The C2f module is designed based on the ELAN module, which obtains more gradient flow branches by paralleling the bottleneck of each layer, ensuring a lightweight structure while obtaining richer gradient flow information. The SPPF module avoids image distortion caused by cropping and scaling operations on image regions, solves the problem of duplicate feature extraction, reduces computational complexity, and greatly improves running speed. The Neck, also known as the FPN-PAN structure, consists of a Feature Pyramid Network (FPN) and a Path Aggregation Network (PAN). The Head adopts Decoupled Head detection technology, which separates the classification branch and regression branch at the output end for training and output, making the model more targeted and improving recognition accuracy.

The basic structure of YOLOv8 consists of three essential parts: (1) The backbone, (2) Neck, (3) Head (See Figure 1). YOLOv8, in its basic form, uses the Mosaic data augmentation technique adopted from YOLOv5. This technique combines and concatenates images (usually four) using a series of other methods, including random scaling, cropping, and arrangement. The use of this technique has several advantages for the learning process. It improves training efficiency and reduces memory consumption. Before the data is fed to the backbone network for processing, the images are first resized to a standard size (usually 640 × 640 pixels). This is done using an adaptive scaling technique to ensure data uniformity.

#### 2.2.1 Backbone network

Fundamentally, the typical backbone network of YOLOv8 consists of convolutional modules (CBS), Cross stage partial Bottleneck with 2 convolution fast (C2f) modules, and fast spatial pyramid pooling (SPPF) modules, ordered systematically to achieve efficient performance. The CBS modules are primarily designed to perform a set of operations, including convolutions by a 2-dimensional convolution layer (Conv2d), batch normalization by a 2-dimensional batch normalization layer (BatchNorm2d), and activation by a SiLU activation layer. And the name CBS stands for these three operations. The C2f modules in the backbone network are responsible for feature extraction, model light weighting, and establishing the bases for further model improvements. The integration of C2f in the backbone network of YOLOv8 improves the feature expression ability of the model and reduces the computation costs of the model. The two convolution operations in C2f modules enable feature extraction, while the cross-stage partial connection facilitates feature fusion. The use of C2f modules can also serve several other purposes, including as a strategy for model lightweighting. For example, early studies (Li and Cheng, 2025; Xie et al., 2024; Zhou X. et al., 2025) have already shown how the number of model parameters and computational costs are significantly reduced by optimizing model with C2f modules (such as C2f-Ghost, C2f-Star, and C2f_LMSMC). Other studies have endorsed the C2f modules as the basis that allows YOLOv8 to flexibly adapt to integration with other technologies. For example, the introduction of the lightweight C2f module (GSConv and partial convolution) to replace the standard convolution and bottleneck structure enabled the model to integrate new backbone networks, such as FasterNet, more flexibly (Guo et al., 2024). Qu and Zhang (2025) demonstrated that integrating C2f with YOLOv8 blocks (StartNet) to form the C2f-star module, further embedded with adaptive fine-grained channel attention (FCA) to build the FS-C2f module, improved the integration ability of YOLOv8 and attention mechanisms (Qu and Zhang, 2025). The SPPF, on the other hand, provides the backbone network with the capability to capture large-scale and fine-grained spatial information at the same time and fuse the features maps at different scales. This capability is referred to multi-scale feature extraction and fusion. The SPPF is optimized, offering the model improved inference speed for better real-time detection performance.

#### 2.2.2 The neck

Studies refer to the neck network of YOLOv8 as the FPN-PAN structure, as it combines the Feature Pyramid Network (FPN) and a Path Aggregation Network (PAN) to achieve efficient fusion and transfer of multi-scale features (Yaseen, 2024; Zhou L. et al., 2025). The FPN part is mainly responsible for enhancing detection capability through up-sampling sections of the model (feature maps) and fusing them via a top-down pathway (Deng et al., 2020; Li et al., 2020; Zhu et al., 2022). The up-sampled high-level features are fused with lower-level features and passed downstream (to the bottom) (Chen et al., 2023; Li et al., 2020; Xie et al., 2023). This process is executed through lateral connections which merge features from different layers, ensuring that the upsampled features are enriched with spatial information from earlier layers before subjecting the information rich in context and detail to the detecting layers or subsequent layers (Zhu et al., 2022).

On the other hand, the core idea in PAN structure in the neck network is to aggregate features or information along specific paths in a network. This process enables a richer, more effective representation by connecting and combining information from different levels or nodes. The PAN enhances feature hierarchies by adding bottom-up paths to complement the traditional top-down flow used in Feature Pyramid Networks (FPN) (Du and Liang, 2024). This process enhances the propagation of low-level features and allows them to be better integrated with high-level semantics (Jin et al., 2022). This integration effectively compensates for the problem of insufficient bottom-up information flow in FPN, which often results in the loss of important details of small or densely packed objects. Thus, the integration of PAN structure results in richer, more informative feature maps that lead to improved detection of small and complex objects (Shi et al., 2024; Sun et al., 2024; Zhang et al., 2023). In essence, the PAN structure uses adaptive feature pooling to connect each feature grid with all feature levels (Zhang et al., 2022). This strategy down-samples the feature maps from different levels (usually low-level features) of the backbone network before aggregating them. This strategic combination ensures that all useful information from all levels is directly available for subsequent processing. By incorporating this strategy, the architecture’s ability for mask prediction and object localization (e.g., identifying the precise location of an object within an image) is significantly improved.

Collectively, the combination of FPN and PAN in the neck network has two significant advantages: first, it addresses the loss of key information during up-sampling in the FPN structure; second, it offers more effective multi-scale feature fusion— capturing both local details and global context—that enhances the model’s ability to detect both large and small objects simultaneously (Jin et al., 2022)

#### 2.2.3 The head

The head of YOLOv8 is the final part of the network, designed to translate feature maps into meaningful bounding boxes and class probabilities. Upon receiving the aggregated feature maps from the neck network, the head of the YOLOv8 splits these features into two branches (parallel paths): one for object classification and the other for bounding box regression using the Decoupled Head detection technique (Chang and Wang, 2024; Chen F. et al., 2024; Xiong et al., 2024). Each branch uses its own set of convolutional layers to learn and emphasize features tailored to its respective task without interference from the others (Li, 2024; Li W. et al., 2024; Qiu et al., 2024). This separation is different from previous versions of YOLO. Wherein parameters for both tasks are shared. In YOLOv8, each task is optimized independently (Chen F. et al., 2024). Such action positively impacts the convergence speed and detection accuracy of the model. The decoupled Head allows the model to work freely with anchor-free detection approach, further reducing the computation complexity (Vasanthi and Mohan, 2024). In short, layers for classification tasks extract features that are highly discriminative for identifying the presence of an object and its class within a potential bounding box. These layers learn to recognize patterns associated with different objects in various categories. Layers for bounding box regression are trained to extract features optimal for determining the precise location of the bounding box around an object, as well as the actual size.

### 2.3 Our improved YOLOv8 network

**Figure 2:**
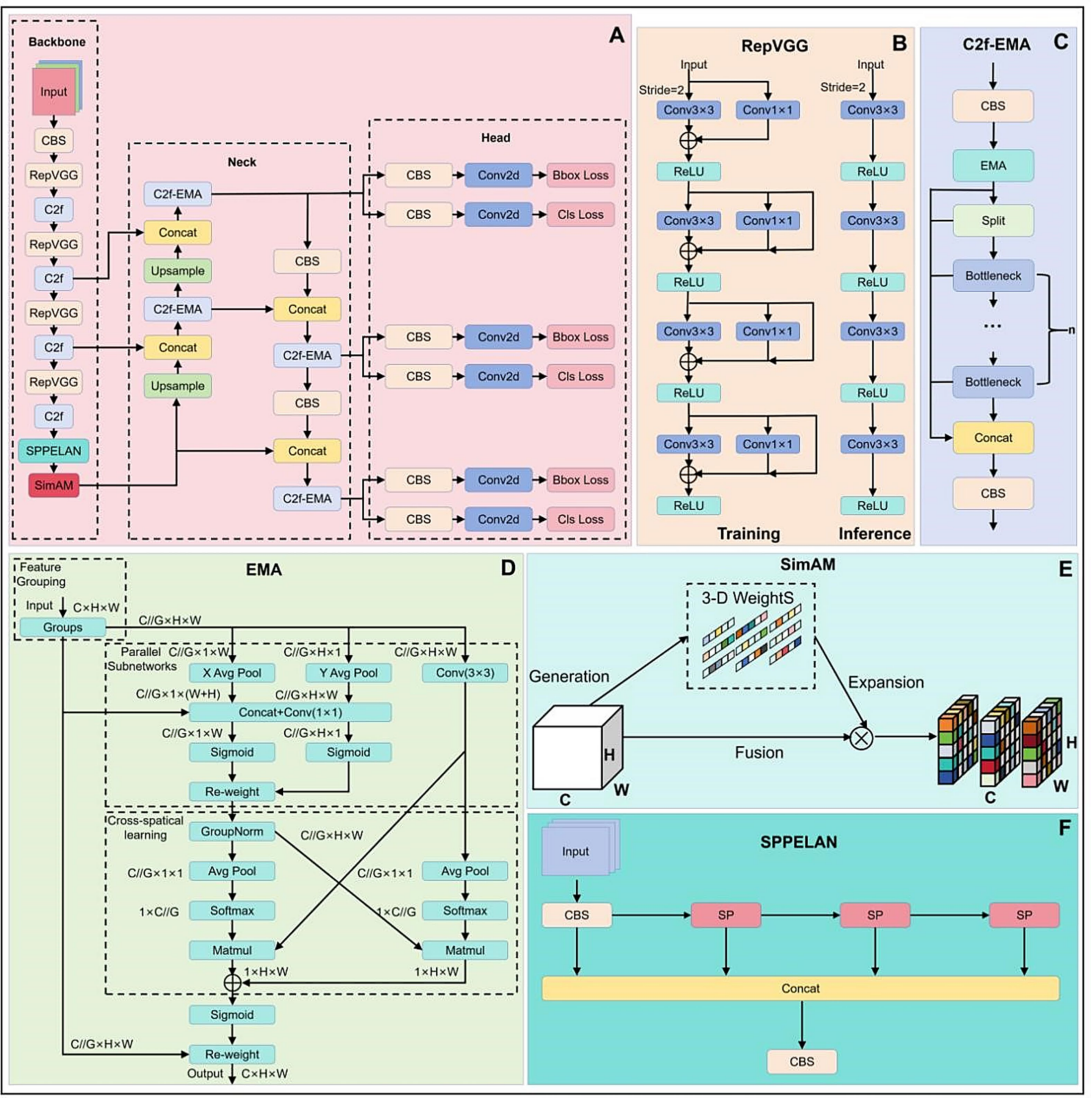
The improved YOLOv8 network model and the specific network structure of the improved parts. We modified and replaced some modules of the original YOLOv8 network model to make it more suitable for MRI image recognition **(A)**. Firstly, in the backbone network, the CBS convolution modules of layers 1, 3, 5, and 7 were replaced with RepVGG modules **(B)** to enhance the training accuracy and inference speed of Alzheimer’s disease MRI medical images; Secondly, the original spatial pyramid pooling layer SPPF is replaced with SPPELAN **(F)**, which enhances the recognition ability of Alzheimer’s disease images by fusing local and global features at multiple scales; Then, SimAM attention mechanism **(E)** was added at the end of the backbone network, enabling the network to better focus on channel attention and achieve feature focus detection of more important and informative features in Alzheimer’s disease images; Finally, some C2f modules in the neck network FPN-PAN were replaced with C2f_EMA modules **(C)**, By adding EMA attention mechanism **(D)** to the C2f module, the input feature weights in the C2f module were dynamically adjusted, enhancing the model’s generalization ability and recognition accuracy for Alzheimer’s disease MRI medical images.

Now we know the basic YOLOv8 architecture. Here we propose significant architectural and modular components to improve the traditional YOLOv8 to serve our diagnostic purpose (See Figure 2).

#### 2.3.1 On the backbone network

##### 2.3.1.1 RepGG

We first introduced the RepVGG (Reparameterized VGG) module to replace the CBS module in the backbone network. The RepVGG is a convolutional neural network tailored to specifically leverage architectural and reparameterization techniques for learning and inference. In the training phase, the use of RepVGG gives the model the ability to utilize a multi-branch strategy. The multi-branch strategy in RepVGG provides access to two types of residual structures: the identity and 1 × 1 branches. This access provides multiple gradient propagation paths for the network, which enhance diversity in gradient flow. In the inference stage, the use of RepVGG offers the OP Fusion strategy, transforming multi-branch convolution operations obtained from the training stage into a 3 × 3 standard convolution layer. The 3 × 3 convolution filters and activation functions stacked together in RepVGG are central to this process. This design reduces computational complexity and the time spent in the inference stage.

##### 2.3.1.2 SPPELAN

Second, we introduced SPPELAN(Spatial Pyramid Pooling Enhanced with ELAN) module to replace the fast spatial pyramid pooling (SPPF). The SPPELAN originates from the original version of Spatial Pyramid Pooling, improved by integrating it with the Efficient Aggregation Network (ELAN). The SPPELAN offers different strategy in performing pooling operations compared to the original SPP. While the SPP employs different window sizes, typically 5, 9, and 13, for these operations, the SPPELAN performs these three operations with the same window size, often 5, to extract features. Concatenation and feature fusion are activated only when all three pooling operations are completed. The introduction of SPPELAN in place of SPP offers several advantages, primarily reducing the number of computational parameters and complexity while achieving similar functionality to SPP. Specifically for our Alzheimer’s data scanned at different scales, the use of SPPELAN may be preferred for improved multi-scale feature extraction and fusion.

##### 2.3.1.3 SimAM

Third, we enriched the end of the backbone network with an additional layer that introduces attention mechanisms. This architectural modification replaces the canonical YOLOv8 backbone end, which is traditionally terminated with the SPP. The use of attention mechanisms enhances the feature expressiveness and the model’s ability to discriminate the features. This technique allows the model to focus (to be attentive) only on the most informative feature regions or channels (Zaryabi et al., 2022; Zhang and Yang, 2021). With this technique in place, the backbone can effectively and dynamically select more relevant or important features and down-weight the less relevant ones. For example, the spatial attention mechanism can guide the network to focus only on object boundaries and key textures, while ignoring other features. On the other hand, the channel attention can emphasize on features that offers high discrimination between classes or categories. These characteristic features of attention mechanisms reduce information redundancy and optimize the flow of information across multiple layers. In essence, the process of identifying the most relevant features or channels involves weigh assignments. The attention module assigns different weights to different features, including spatial location, channels, or elements. The weights of the features are then updated during backpropagation. The adjustments of the weights depend on how they influence the outcomes or minimize the loss function. It is important to understand that information from the backbone network comes as a mixture of low-level and high-level features. However, only the richer and more informative features are needed for the subsequent layers of the model for further processing. This is where attention mechanisms come into play. In this case, attention mechanisms are needed to determine how much emphasis should be placed on fine-grained details versus semantic information to achieve desired outcomes. The entire process boosts the model’s learning speed and convergence. For the task at hand, the use of attention mechanisms can effectively improve the detection of AD without increasing the parameters.

#### 2.3.2 On the neck network

##### 2.3.2.1 C2f-EMA

We also modified the neck network of YOLOv8 by replacing the C2f with a variant called C2f-EMA. The C2f-EMA integrates two technologies: C2f and Efficient Multi-scale Attention (EMA). This integration addresses the issue of the C2f module being insensitive to small objects, complex backgrounds, and multi-scale information. The C2f-EMA can better capture fine-grained features in small targets and local features, reducing the rate of missed and false detections often experienced by the sole C2f module. The dynamic approach of EMA in assigning weights to input features enables the network to automatically identify the most relevant local features. The EMA attention in C2f-EMA propagates information across space and across channels, enhancing the model’s ability to fuse multi-scale features. The EMA mechanisms are also embedded with a channel-reshaping strategy, allowing for the characterization of channels into multiple sub-feature groups. These mechanisms offer the additional advantage of model lightweighting by reducing the number of model parameters and calculations.

### 2.4 Data organization and preprocessing

In this study, we utilized the MRI data from open-source Alzheimer’s disease brain MRI image database offered by universe.roboflow.com. A total of 1,592 images were recruited. Of these 1,592 images, 400 were from individuals without dementia, 400 from individuals with mild dementia, 399 from individuals with moderate dementia, and 393 from individuals with severe dementia. Each image was standardized to 640 × 640 pixel size. We split the data into three groups, each with a specific task. The first group consisted of 70% (1,115 of 1,592) of the total data and was used for training the model. The second group consisted of 20% (319 of 1,592) of the total data and was used for validation. The third group had 10% (158 of 1,592) of the total data and was reserved for testing the model.

### 2.5 Environment setup and model training

The environment for data processing and model training was created Linux Server System, equipped with GPUs (NVIDIA GeForce RTX 4090, 24BG memory). This environment was created by Python v3.9, with supporting packages including CUDA v11.8 and Pytorch v2.5.1. We trained our model with 64 batch size and 400 epochs. This choice was made to ensure sufficient training. The training was performed with a learning rate of 0.01 and the cosine adjuster (cos_lr) enabled.

### 2.6 Model evaluation and strategy

The model was evaluated using four metrics: Precision (P), Recall (R), Average Precision (mAP50, IOU = 0.5), and mAP50:95(IOU range, 0.5 to 0.95). Each metric evaluates specific aspect of the model. The precision quantifies the percentage of true samples predicted as true positives in the overall samples predicted as positive. In a nutshell, this metric assesses how many were truly positive among those predicted to be positive. Recall measures the percentage of true positives predicted as positives among all actual positive samples. On the other hand, the other measures mentioned evaluate the overall performance of the model. We also estimated parameter quantity, which quantifies the total number of parameters used in the model. This parameter reflects the size and complexity of the model. To evaluate the impact of each modification to the model, we adopted a unit-by-unit ablation approach (removing a module and replacing it with a new one) and observed model performance before assessing collective impact of all modifications.

## 3 Results

### 3.1 Performance by modifications

**Figure 3:**
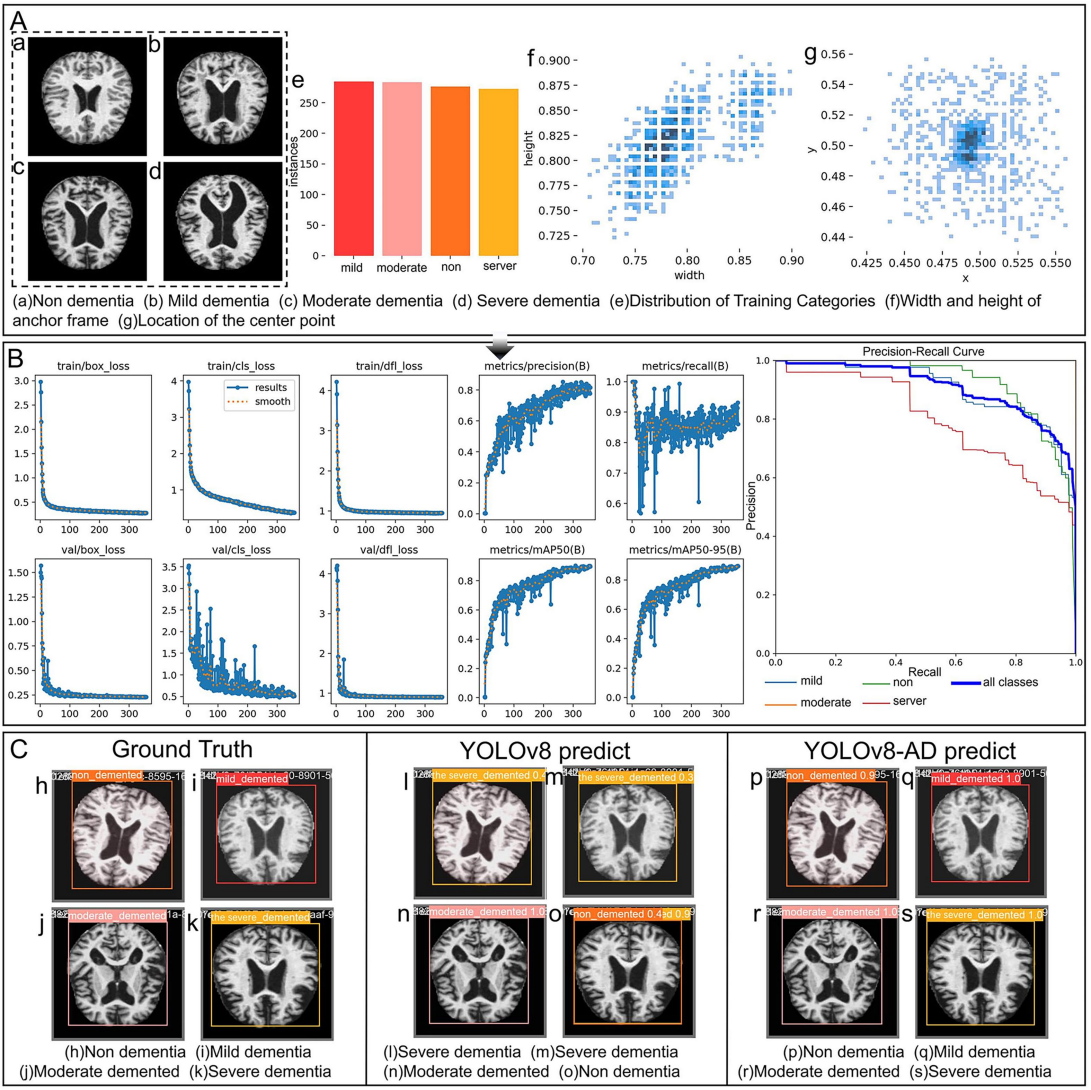
Model evaluation and characterization. The nature of the data fed to the model is shown in **(A)**. Four types of data were organized: (a) Non demantia, (b) Mild dementia, (c) Moderate dementia and (d) Severe dementia. Before training, the data were distributed as shown in (e), with the anchor frame defined by width and height in (f), with the center point (g). The performance of our improved model architecture is described and shown in **(B)**. Briefly, our improved architecture reached the following performances as described by different metrics: 0.816 in precision, 0.877 in recall, 0.904 in mAP50, and 0.898 in mAP50:95. The typical recognition of images per category is shown in **(C)**.

Table 1 summarizes the model’s performance under various modification conditions and ablation settings (removing and replacing some modules) (also see Figure 3). Generally, modifying the backbone by replacing CBS with RepVGG improved the model’s performance by 0.6%, from 87.5 to 88.1% in Map50:95 measures. Modifying the model by only introducing simAM at the backbone network provides an improvement of 0.7% as well (from 87.5 to 88.2% in mAP measures). There was no significant change in performance when SPPELAN was introduced to replace the canonical SPP (87.5% with SPP and 87.8% with SPPELAN). Nevertheless, the modification of the neck network by C2f-EMA significantly improved the model. There was an increase in model performance by 1.4% (from 87.5% in C2f to 88.9% in C2f-EMA). Of interest is that the model performance offered by SimAM and C2f-EMA is similar to the effects offered by SimAM and canonical C2f (88.2% with SimAM and C2f-EMA, and 88.2% with SimAM and C2f, mAP measures). The best results were observed when all aspects were modified simultaneously. The model performance improved by 2.7% in mAPs(50:95), from 87.5 to 89.8%, following these modifications. These improvements, quantified by mAP50:95, were in line with those observed in mAP50. The mAP50 results demonstrated that these modifications in the model raised the value of this metric to 90.4% from 88.1%. These results also underscore that mAP can be a better proxy measure of model performance in detection problems than literal estimates from precision and recall. This is evident from our study, where the recall values alone seem unstable or appear to fluctuate as the model undergoes modification. The highest recall performance occurs when RepVGG replaces CBS, while the highest precision performance is observed when C2F-EMA is introduced in the neck network.

**Table 1:** Performance of YOLOv8 model architecture across different module adjustments.

Index	YOLOv8	RepVGG	SimAM	SPPELAN	C2f_EAM	*p*/%	R/%	mAP50/%	mAP50:95/%	Parameters
1	√					0.794	0.876	0.881	0.875	3,006,428
2	√	√				0.77	0.91	0.887	0.881	3,051,388
3	√		√			0.827	0.84	0.888	0.882	3,006,428
4	√			√		0.78	0.889	0.884	0.878	3,170,396
5	√				√	0.862	0.837	0.895	0.889	3,022,716
6	√		√		√	0.828	0.86	0.889	0.882	3,022,716
7	√		√	√	√	0.824	0.817	0.882	0.876	3,186,684
8	√	√	√	√	√	0.816	0.877	0.904	0.898	3,231,644

### 3.2 Performance by models

When compared with other models (Table 2), our model offers better performance. While our model achieved a performance of 90.4% by mAP50 measures and 89.8% by mAP50:95 measures, the other models had relatively lower performances (88.1% mAP50 and 87.7% mAP50:95 for YOLOv5; 85.8% mAP50 and 85.3% mAP50:95 for YOLOv6; 88.1% mAP50 and 87.5% mAP50:95 for YOLOv8).

**Table 2:** Comparison of model performances.

Model	*p*/%	R/%	mAP50/%	mAP50:95/%	Parameters
YOLOv5	0.849	0.831	0.881	0.877	2,503,724
YOLOv6	0.771	0.856	0.858	0.853	4,234,140
YOLOv8	0.794	0.876	0.881	0.875	3,006,428
YOLOv8-AD	0.816	0.877	0.904	0.898	3,231,644

## 4 Discussion

For the first time, this study underscores that the detection of Alzheimer’s disease may be improved when either of the following aspects are modified or introduced: (1) RepVGG, (2) simAM, and (3) C2F-EMA. The improvements seen after introducing RepVGG suggest that RepVGG plays a significant role in enhancing training and detection performance. This ability of RepVGG has been explained previously, with studies emphasizing that its use boosts model performance due to improved multi-scale feature learning (Li and Liang, 2024; Zhang, 2023) and feature extraction ability, and faster inference speed through a simplified computational graph in the network inference stage (Li et al., 2023; Wang et al., 2024). Similar to our study, several other studies have shown improvements in the mAP metric after replacing CBS with RepVGG. For example, Zhang et al. (2023) reported an improvement of around 13.4 to 17.4% on the VisDrone2019 dataset after the replacement (Zhang, 2023). On the other hand, while Li et al. (2023) reported an improvement of 3.8% after this replacement for small object detection (Li et al., 2023; Li et al., 2024) showed a 2.2% rise in performance in early fire detection problems (Li and Liang, 2024). Taken together, these early studies, in conjunction with ours, emphasize the importance of RepVGG in improving the ability to perform complex tasks, including AD detection, and suggest that RepVGG capability is not constrained to a few areas but is generalizable.

The improvements observed after introducing simAM may reflect the potential advantages of attention mechanisms. It is now clear that simAM significantly contributes to the improvement of model performance. Studies have repeatedly demonstrated the ability of attention mechanisms to learn by focusing on both spatial and channel-wise details. Since the detection task requires the combination or fusion of multi-scale features, attention mechanisms provide this pathway more effectively by not only obtaining these features but, more importantly, by selecting the most relevant ones while suppressing noise features and improving the robustness and generalization ability of the model. To be more specific, studies have shown an improved mAP of 0.8 to 9.5% after integrating simAM modules into their models (Chen et al., 2025; Guo et al., 2024; He et al., 2024; Li N. et al., 2024; Yuan et al., 2025). And it seems that the simAM offers this advantage without any additional need of model parameters or computations (Guo et al., 2024; Han et al., 2025; Han et al., 2024; Luo et al., 2023; Zhu G. et al., 2024). In our case of AD, the use of simAM enhances the capturing of lesion areas that are small and scattered through adaptive feature enhancement and suppression of irrelevant background information, making it easy to detect the AD-related subtle changes.

Our study also provides evidence that replacing C2f with C2f-EMA improves model performance, particularly in the detection of Alzheimer’s disease from MRI images. This improvement suggests that the C2f variant, especially those incorporating efficient multi-scale attention mechanisms, enhances the model’s efficiency. For example, in underwater garbage detection, there was a significant improvement of about 6.7% in accuracy, 4.1% in recall, and 5% in mAP following the introduction of C2f-EMA (Zhu J. et al., 2024). Other works include those by Lu and Liuai (2023) and Chen et al. (2024) endorsed C2f-EMA as more effective to traditional C2f (Chen J. et al., 2024; Lu and Liuai, 2023).

Although we observed independent improvement from each adjustment or technology introduced, our study advocates the importance of combining different technologies for more significant and synergistic improvement than that which could be obtained by a single technology. Combining multiple technologies such as RepVGG, SimAM, SPPELAN, and C2f-EMA in the same model, rather than using a single technology, can lead to more comprehensive and significant performance gains. The fusion of multiple technologies can achieve synergistic gains in terms of accuracy, recall, and object detection capabilities, which is significantly better than a single technology. Similar thoughts have been clarified before (He et al., 2024). It is important to note that each technology has a specific aspect it improves and is as important as the other aspects. For example, RepVGG helps the model detect complex and dense targets, even as small and dense as weeds. Attention mechanisms such as simAM and EMA help distribute focus on the features, identifying which ones are more relevant than others. These attention mechanisms navigate dynamically across both local and global information of an image or feature map to identify the importance of each aspect of feature in the detection of AD pathology. On the other hand, technologies such as C2f offer high sensitivity to large morphological changes and shapes, even needle-like shapes (He et al., 2024). At the same time, SPPF technology provides a powerful capability for multi-feature adaptation during feature fusion, unifying non-uniform, multiscale features into a uniform feature representation. This enables effective feature learning and detection without worrying about variations in scales, lesions, shapes, or levels of morphometric changes or atrophy experienced by regions such as the hippocampus or ventricles due to AD pathology.

Originally, YOLO models were designed for real-time detection of objects in images and videos. The initial applications were mostly in computer vision tasks, aiming to fill the gap by having fast/rapid, real-time and accurate model for object detection, including identifying people, vehicles and animals in an image or video. This identification were targeted for surveillance and autonomous driving. Nevertheless, recently, YOLO models have been adopted for medical image analysis tasks such as diagnosing diseases like AD, lesion detection, tumor segmentation, and identifying abnormalities in various organs (Huang et al., 2025; Liu, 2022; Ragab et al., 2024). From late 2010 to early 2020, significant efforts have been made to explore its potential in AD diagnosis, with a focus on detecting brain regions related to the disease. The YOLO models’ ability to process images quickly and accurately has made it valuable for computer-aided diagnosis, outperforming some traditional methods in speed and precision. This enables the expansion of its application to the detection of lung nodules, skin lesions, retinal abnormalities, cardiac issues, and brain tumors (Huang et al., 2025; Liu, 2022; Ragab et al., 2024).

### 4.1 Limitations

This work is part of efforts to explore the potential of the YOLO model series for detecting AD. The authors acknowledge that there are still limitations needing improvement. Some limitations include the use of MRI medical images from a single modality; therefore, there is a need for multi-target details, which may include information from other imaging modalities connected for multi-model detection. This would offset modality-specific limitations likely to confound model performance while leveraging each modality’s potential in the detection process.

## 5 Conclusion

Here we conclude that with advancements in model architecture design and mechanisms to enhance effective training, as well as specific data extraction and fusion, the goal of improving early detection of complex conditions like Alzheimer’s disease can be achieved. We have specifically seen that there is a strong potential for YOLOv8 in these tasks. We have seen improvements in the backbone network by RepVGG, SPPELAN, and SimAM significantly and independently enhancing model performance, as evidenced by mAP50 and mAP50:95% metrics. Our findings also demonstrate that further integrating attention mechanisms in the neck network by replacing C2f with C2f-EMA can synergistically boost the overall performance of the model to about 90.40% mAP50 from 88.10% mAP50 in the canonical YOLOv8. Taken together, these findings provide hope that further improvements to the model can enable the model achieve its maximum efficiency, enabling its deployment as a diagnostic tool for early AD, which is essential for initiating early intervention.

## Data Availability

The raw data supporting the conclusions of this article will be made available by the authors, without undue reservation.
